# Inducible metabolic pathway for citrate metabolism in case of major liver dysfunction: fact or fiction?

**DOI:** 10.1186/s13054-019-2432-7

**Published:** 2019-05-14

**Authors:** Patrick M. Honore, David De Bels, Sebastien Redant, Rachid Attou, Luc Kugener, Willem Boer

**Affiliations:** 1 ICU Department, Centre Hospitalier Universitaire Brugmann, 4,Place Van Gehuchten, 1020 Brussels, Belgium; 2 Department of Anesthesiology, Intensive Care Medicine, Emergency Medicine & Pain Medicine, Ziekenhuis Oost-Limburg, Genk, Belgium

With interest, we read the paper published by Klingele et al. [1] in a recent issue of critical care.

Because citrate metabolism is oxygen dependent via the citric acid cycle and therefore mainly takes place in organs with high amounts of mitochondria, such as the liver, kidney, or muscle [1], they hypothesized that adequate citrate metabolism must be possible outside the liver, implying an inducible metabolic pathway. They postulate that disturbed microvascular circulation could cause altered hepatic function, resulting in the association of impaired liver function and citrate accumulation.

In neonates with mitochondrial cytopathy [2], the citric acid cycle does not function properly and therefore anticoagulation with citrate is contraindicated. We report two cases of neonates suffering from mitochondrial cytopathy in whom unfractionated heparin was stopped because of bleeding complications. Circuit half-lives without anticoagulation were short (8 h). To remedy this, it was decided to introduce citrate slowly but progressively, increasing the dose, monitoring for accumulation every 2 h. After 96 h, the two neonates were able to metabolize citrate despite citric acid cycle dysfunction, inferring activation of the inducible pathway.

It is well known that in all cells, citrate can be metabolized within the Cori cycle (tricarboxylic acid cycle) [1]. It describes the linked metabolic pathways by which muscles, even in the absence of oxygen, remain capable of functioning, producing lactate. It is activated both by citrate [2] and adrenaline, the latter leading to the production of lactate [3]. The muscles normally combine glucose with oxygen to generate energy. In anaerobic circumstances, lactate is produced which is synthesized by the liver through gluconeogenesis, the process of glucose production from non-carbohydrate components.

In the setting of citrate anticoagulation, with disturbed microvascular circulation causing altered hepatic function and citrate accumulation [1], it is also our experience that lactate better reflects citrate accumulation than calcium ratio and liver dysfunction itself. However, in a recent review, Schneider et al. concluded the opposite [4]. For them, lactate elevation is not secondary to citrate accumulation but due to a common primary process impairing the tricarboxylic acid cycle, reducing citrate metabolism, and limiting pyruvate metabolism leading to lactate generation [4]. The liver citrate anticoagulation threshold (L-CAT) trial showed the safety of continuous renal replacement therapy (CRRT)- citrate in patients with severely impaired liver function [5]. We conclude that Cori cycle is functional without oxygen [2] and that citrate metabolism is less dependent on the hepatic function itself and more dependent upon the whole microcirculation of the body as suggested by Klingele [1].

## Abbreviations

CRRT: Continuous renal replacement therapy
L-CAT: Liver citrate anticoagulation threshold
