# Supplementary material for: Inducible metabolic pathway for citrate metabolism in case of major liver dysfunction: fact or fiction?
Source: Crit Care. 2019 May 14;23:166. doi: 10.1186/s13054-019-2432-7 (PMC6515620; doi:10.1186/s13054-019-2432-7)
Supplement: Related texts

# Reply: Authors’ response

**Authors:** Matthias Klingele, Theresa Stadler, Danilo Fliser, Timo Speer, Heinrich V. Groesdonk, Alexander Raddatz

Honore and colleagues report about two neonates suffering from mitochondrial cytopathy being able to metabolize citrate despite citric acid cycle dysfunction. They conclude from this interesting observation that this infers activation of an inducible pathway, and they refer to our study [1].

We reported about long-term continuous renal replacement therapy (RRT) and anticoagulation with citrate (RCA) in critically ill patients with severe liver dysfunction and unexpected low rates of citrate accumulation. Thus, the citrate metabolism in extrahepatic organs with high amounts of mitochondria such as the muscles was mostly sufficient to deal with the amount of citrate delivered by CRRT with RCA. We therefore concluded that a substantial extrahepatic metabolism of citrate seems to exist. However, in case of disturbed microcirculation, both hepatic and extrahepatic citrate metabolisms were clearly disturbed [1]. In a systematic review, Zangh et al. confirm our findings. They could demonstrate that patients with and without liver dysfunction showed similar laboratory parameters during CRRT with RCA, indicating a comparable citrate metabolism in patients with and without liver dysfunction [6].

Our hypothesis was that the risk of citrate accumulation during regional citrate anticoagulation is more dependent on microcirculation than on liver function. Disturbed microcirculation results in elevated lactate due to impaired organic substrate metabolism. Therefore, lactate is a risk factor for impaired citrate metabolism. This is supported by Khadzhynov and colleagues [7]. However, they suggest that lactate kinetics rather than initially elevated lactate concentration should be considered in assessing the risk of citrate accumulation.

Honore and colleagues agree that disturbed microvascular circulation and lactate are a hint for citrate accumulation and that extrahepatic citrate metabolism explains why CRRT with RCA in case of liver dysfunction not necessarily results in citrate accumulation. Moreover, they suggest an inducible pathway which can be activated despite mitochondrial cytopathy and citric acid cycle dysfunction. This is an interesting and novel approach, being able to explain why hepatic function may be less important for citrate metabolism in CRRT with RCA than previously thought.
